# Gut Microbiota: An Integral Moderator in Health and Disease

**DOI:** 10.3389/fmicb.2018.00151

**Published:** 2018-02-21

**Authors:** Qingqing Feng, Wei-Dong Chen, Yan-Dong Wang

**Affiliations:** ^1^State Key Laboratory of Chemical Resource Engineering, College of Life Science and Technology, Beijing University of Chemical Technology, Beijing, China; ^2^Key Laboratory of Receptors-Mediated Gene Regulation and Drug Discovery, School of Medicine, Henan University, Kaifeng, China; ^3^Key Laboratory of Molecular Pathology, School of Basic Medical Science, Inner Mongolia Medical University, Hohhot, China

**Keywords:** gut microbiota, miRNA, intestinal disease, liver disease, lung

## Abstract

The gut microbiota, as the main member in gut microecology, is an essential mediator in health and disease. The gut microbiota interacts with various organs and systems in the body, including brain, lung, liver, bone, cardiovascular system, and others. Microbiota-derived metabolites such as the short chain fatty acid (SCFA) butyrate are primary signals, which link the gut microbiota and physiology. Recently, the gut microbiota has been identified as the origin of a number of diseases by influencing the related cell signaling pathways such as WNT/beta-catenin pathway in colorectal cancer and T cell receptor signaling in the central nervous system. Moreover, several microRNAs participate in signaling networks through the intervention of the gut microbiota. The interaction between the gut microbiota and miRNAs plays a crucial role in vascular dysfunction and hepatocellular carcinoma (HCC). In this review, we will report and discuss recent findings about the crosstalk between the gut microbiota and physical organs and how the gut microbiota and miRNAs regulate each other while influencing the host via genes, proteins, or metabolites.

## Introduction

Growing investigations on host–microbe interactions have revealed that the gut microbiota is a critical mediator in maintaining health (Holmes et al., 2011). The “gut microbiota” generally refers to the diverse microbial community (bacteria, fungi, archaea, viruses, and protozoa) that colonizes their host’s gastrointestinal tract (Shukla et al., 2017). The composition of gut microbiome is similar at the phylum level (mainly *Bacteroidetes* and *Firmicutes*), but diversity and richness of species is variable between individuals (Tremaroli and Backhed, 2012). Host genetics, environmental factors, diet, disease, stress, and some other factors decide the structure of the gut microbiota (Shukla et al., 2017) while the microbiota dictates host’s health and diseases via genes, proteins or metabolites (Van de Wiele et al., 2016).

Physiological energy homeostasis could be regulated by microbiota. For example, Donohoe et al. (2011) reported that microbiota-produced butyrate could provide energy to colonocytes and then prevent autophagy in the colon. In addition, physiological homeostasis may be disrupted by the microbiota, resulting in disruption of host metabolism, immune dysregulation, neurological and cognitive dysfunction and others (Roy and Trinchieri, 2017). This disrupted status would cause a series of disorders, including obesity, diabetes, autoimmunity, allergy, inflammatory bowel disease (IBD) and cancer (Holmes et al., 2011; Buret, 2016). Emerging data of the gut microbiota reveal that the microbiota is involved in diverse diseases via gut–brain axis (Baruch and Schwartz, 2016; Mu et al., 2016; Dinan and Cryan, 2017; Kennedy et al., 2017), gut–lung axis (Budden et al., 2017; Shukla et al., 2017), gut–liver axis (Nicolas et al., 2017; Xue et al., 2017), gut–bone axis (Ohlsson and Sjogren, 2015; Yan et al., 2016; Ridler, 2017; Villa et al., 2017), gut–vascular axis (Zhu et al., 2016; Li et al., 2017), and other axes.

MicroRNAs (miRNAs) are regarded as regulators of various diseases (Rupaimoole and Slack, 2017). It has been found that microbiota-produced butyrate regulated hepatic cell apoptosis and proliferation by inducing miR-22 expression (Pant et al., 2017). The research about gut microbiota–miRNA interaction has revealed that the gut microbiota could be regulated by host-secreted miRNAs and the gut microbiota may affect the host via inducing miRNAs (Liu et al., 2016; Belcheva, 2017). The gut microbiota–miRNAs-diseases axis could serve as a new direction for future investigation.

In this review, we summarize the physiological and pathological functions of the gut microbiota including signals from gut to other organs. Specifically, we highlight the new functions about the gut microbiota in diseases and how the gut microbiota monitors the host status by governing special miRNAs.

## Microbe–Host Communication in Disease

Since the gut microbiota settles in gastrointestinal tract, it is unsurprising that microbiota contributes to regulating intestinal diseases (Holmes et al., 2011). However, emerging evidence indicates that the microbiota is implicated in extraintestinal organs due to the difference of microbiota composition. This altered microbiota composition might be attributed to diet or the environment (cold, antibiotics, use of probiotics, and the occurrence of infections) and dominate the host’s physiological state. More substantially, communication pathways between the gut and other organs are mediated by direct neuronal contact, enteroendocrine cells, immune cells and microbial metabolites (Schroeder and Backhed, 2016). Part of cell signaling pathways in the process has been presented in **Table 1**. It is pivotal to explore how the microbiota communicates with host and develops disease. These relevant investigations will be revealed as follows.

**Table 1:** Gut microbiota influences cell signaling pathways in diseases.

Disease	Signal molecular derived from gut microbiota	Cell signaling pathways	Reference
Colorectal cancer	Butyrate	NF-κB MAPK Wnt/β-catenin	Stempelj et al., 2007; Scott et al., 2008; O’Keefe et al., 2009; Zhang et al., 2010; Fung et al., 2012; Yang et al., 2013; Zeng et al., 2014
	LPS	T cell receptor signaling ERK1/2	Zhu et al., 2011
	Lipoteichoic acid (LTA)	TLR2 signaling	Yang et al., 2013
CNS	GABA	Synaptic signaling	Barrett et al., 2012; Foster and McVey Neufeld, 2013; Kennedy et al., 2017
	SCFAs	Dopamine signaling in Parkinson’s disease	Sampson et al., 2016
	Amyloids and LPS	Amyloid plaque and neurofibrillary tangle formation in Alzheimer’s disease	Köhler et al., 2016
	Dopamine, serotonin (5-HT), neuropeptide, substance and vasoactive intestinal peptide	T cell receptor signaling	Powell et al., 2017
Liver disease	LPS	LPS-TLR4 signaling	Xue et al., 2017
	ND	FXR signaling Bile secretion pathway	Sayin et al., 2013
	Butyrate	PI3K/Akt signaling Mitochondrial control of apoptosis	Pant et al., 2017
Lung disease	LPS SCFA	T cell receptor signaling	Trompette et al., 2014; Thorburn et al., 2015; Budden et al., 2017; Gray J. et al., 2017; Tamburini and Clemente, 2017
	Polysaccharide A		
Vascular dysfunction	LPC18:1	AKT	Li et al., 2017
	SCFAs	MCP-1/CCR-2 signaling IL-17 signaling	Karbach et al., 2016
Osteoporosis		PI3K/AKT/mTOR	Bakker et al., 2016
	SCFAs	T cell receptor signaling	Ohlsson and Sjogren, 2015


*ND, no data available.*

### Gut Microbiota and Intestinal Disease

The gastrointestinal tract is an extremely complex organ system. In symptom, dysbiosis of the gut microbiome forms part of the etiology of various gastrointestinal diseases, especially colorectal cancer (CRC) and IBD (O’Keefe et al., 2009; Zhu et al., 2011; Ohigashi et al., 2013; Yang et al., 2013; Gao et al., 2015; Yuan et al., unpublished). De Filippo et al. (2010) investigated the gut microbial composition from European children (a modern western diet) and Africa children (a rural diet with high fiber) and found a significant abundance of *Bacteroidetes* and a significant decrease of *Firmicutes* in Africa children compared with European children. In parallel, Enterobacteriaceae (*Shigella* and *Escherichia*) were higher in European children than that in Africa children (De Filippo et al., 2010). Nucleotide-binding oligomerization domain-containing protein 2 (one of the genes associated with IBD) risk allele count increases with Enterobacteriaceae relative abundance, which may well explain the higher occurrence of IBD in Europe than that in Africa (Farrokhyar et al., 2001; Knights et al., 2014). Intestinal microbial composition is also a common factor in CRC. Gao et al. (2015) reported that the levels of *Firmicutes* and *Fusobacteria* were higher, while the abundance of *Proteobacteria* was decreased in CRC patients (Gao et al., 2015). Members of *Fusobacteria* has been identified as pro-inflammatory via recruiting myeloid-derived tumor-infiltrating immune cells such as tumor-associated macrophages (TAMs), dendritic cells (DCs), and myeloid-derived suppressor cells (MDSCs) (Kostic et al., 2013; Patel et al., 2016).

In addition, the gut microbiota affects the host via the immune system or metabolites. Short chain fatty acid (SCFA) butyrate, as a histone deacetylase (HDAC) inhibitor, is the most commonly studied metabolite. Ohigashi et al. (2013) reported that SCFAs (acetic acid, propionic acid, and butyric acid) were significantly decreased in CRC group (Ohigashi et al., 2013). Numerous studies also confirmed butyrate is involved in several signaling pathways in CRC (**Table 1**), including suppressing nuclear factor-kappa B (NF-κB), inducing WNT/beta-catenin activity and apoptosis, activating mitogen-activated protein kinase (MAPK) signaling pathway by upregulation of GADD153 or activation of phosphorylation of c-jun N-terminal kinase (JNK) (Scott et al., 2008; O’Keefe et al., 2009; Zhang et al., 2010; Fung et al., 2012; Yang et al., 2013; Zeng et al., 2014). In addition, lipoteichoic acid (LTA) produced by the gut microbiota, a TLR2 ligand, promoted inflammation in host. However, LTA-deficient *Lactobacillus acidophilus* inhibited inflammation and could prevent against colon cancer, colitis and polyposis (Yang et al., 2013). Overall, paying attention to the roles that the gut microbiota plays in regulating immune responses and tumorigenesis in the gastrointestinal tract will be necessary for disease prevention.

### Microbiota–Gut–Brain Axis in CNS

Adaptive immunity (especially T cells) and innate immune system contribute to gut–brain communications via regulation of immune activity and the production of proinflammatory cytokines in IBD, irritable bowel syndrome (IBS) and functional dyspepsia (Leue et al., 2017; Powell et al., 2017). Alzheimer’s disease (AD) is a form of dementia associated with aging, and signaling pathways of gut–brain axis participate in the disease. The microbiota composition changes due to aging and environmental factors, while function of the intestinal mucosal barrier reduces, and bacterial amyloids and lipopolysaccharides (LPSs) systemically leak with increasing age. These toxins are translocated to the CNS due to the increase of blood–brain barrier permeability associated with aging. At the same time, gut dysbiosis affects amyloid beta peptide physiology possibly by changing energy metabolism and insulin resistance. LPS/amyloids increases microglial activation through TLRs-mediated inflammatory response, induces inflammation, and reduces the phagocytic clearance of amyloid. Subsequent inflammation would lead to the onset/progression of neurodegeneration and amyloid beta peptide accumulation (Köhler et al., 2016). Moreover, in a model of Parkinson’s disease, the gut microbiota could regulate motor deficits and neuroinflammation via SCFAs. SCFAs modulate *α*-synuclein-induced microglia activation in the brain and promote *α*-synuclein-dependent neuroinflammation and motor dysfunction (Sampson et al., 2016) (**Table 1**). Microbial metabolites produced by an imbalanced microbiota aggravated and participated in the pathogenesis of certain neurologic conditions. Accurate identification of relevant flora and metabolites may benefit from the discovery of new drug targets. Currently, very limited data about the interactions of the microbiota–gut–brain from human studies exist. The roles of the microbiota–gut–brain axis in CNS are highly plausible. New animal and clinical studies may provide novel approaches for prevention and treatment of mental illness (Fung et al., 2017).

### Gut Microbiota and Liver Disease

The gut microbiota may modulate alcohol liver disease (ALD), non-alcoholic fatty liver disease (NAFLD), cirrhosis, and even hepatic carcinoma (Betrapally et al., 2017). NAFLD has been the extremely frequent origin of chronic liver disease with growing obesity. Probiotics participate in obstructing the development of NAFLD via suppression of the LPS-TLR4 signaling pathway (Xue et al., 2017). Hepatic gluconeogenesis could be controlled by the gut microbiota. Probiotics induced hepatic gluconeogenesis while caecal microbiota from obesity reduced markers of hepatic gluconeogenesis (Nicolas et al., 2017). Recent report showed the mechanistic link between microbiota and hepatocellular carcinogenesis (Xie et al., 2017). The report revealed that sex-based disparity in liver carcinogenesis is associated with the gut microbiota, bile acids, and tumor-suppressive microRNAs (miR-26a, miR-26a-1, miR-192, miR-122, miR-22, and miR-125b) in male and female mice treated with the streptozotocin-high fat diet (STZ-HFD). Microbiota regulated bile acids and microRNAs promoting the hepatocellular carcinoma (HCC) in a male mouse model, however, the regulatory mechanism is unclear. Elevated levels of farnesoid X receptor (FXR), a bile acid nuclear receptor, in female mice may increase expression of miR-26a, miR-26a-1, and miR-122, as the suppressors in liver cancer, possibly resulting in a lower risk of developing liver cancer in female mice (Xie et al., 2017). Additionally, butyrate from microbiota induces apoptosis through up-regulation of miR-22 expression and repression of sirtuin1 (Sirt-1) expression in hepatic cells (Pant et al., 2017). FXR plays a pivotal role in host liver metabolism, including liver regeneration, hepatoprotection, prevention of NAFLD and hepatocarcinogenesis (Wang et al., 2008a,b; Chiang, 2013; Zhang et al., 2016). Zhang et al. (2016) reported that modulation of the gut microbiota by inhibition of intestinal FXR signaling alters host liver lipid metabolism and improves obesity-related metabolic dysfunction. Overall, FXR, miRNA, and SCFAs were relevant mediators of the gut’s microbiota participation in liver disease.

### Microbiota and Gut–Lung Axis

The study about gut–lung axis is in the initial stage, but it may potentially serve as a new direction for lung disease treatment (Budden et al., 2017). Asthma is a relatively stubborn bronchial disease. In childhood asthma, it may be closely related to decreasing relative abundance of the genus *Faecalibacterium*, *Lachnospira*, *Veillonella*, and *Rothia* and altered metabolites (Arrieta et al., 2015). Microbiota-accessible carbohydrates (particularly dietary fermentable fiber) can shape the lung immunity via changes in the microbiota and increase in SCFAs (Gray L.E. et al., 2017). Previous reports show that SCFAs may stimulate Tregs to protect against airway inflammation via activation of GPR43 (acetate and propionate) or inhibiting HDAC (butyrate) (Trompette et al., 2014; Thorburn et al., 2015; Gray L.E. et al., 2017). Dietary fermentable fiber leads to changes in the ratio of *Firmicutes* to *Bacteroidetes* and SCFA production. Propionate is capable of improving the bone marrow hematopoiesis of DC precursors. Furthermore, these DCs were in a position to stimulate T helper type 2 effector cells in the lung. The propionate-mediated mechanism may well protect lung from allergic airway inflammation and this process depends on GPR41 (Trompette et al., 2014). Trompette et al. (2014) found that high-fiber promoted the increase of acetate, leading to silencing transcription of Foxp3 genes in the lung, which were characterized by enhanced T-regulatory cells in numbers and function, and the process might lead to inhibition of allergic airways disease (a model for human asthma) (Thorburn et al., 2015). Gray J. et al. (2017) reported the interaction between the host immune system and the intestinal commensal bacteria. The colonization of gut commensal bacteria promotes the capability of IL-22-producing ILC3s (IL-22^+^ILC3) in preferential trafficking to the lung, inducing IL-22 production and expression of the lung homing signal CCR4. Finally, it promotes the newborn’s IL-22-dependent resistance to pneumonia (Gray J. et al., 2017; Tamburini and Clemente, 2017). In summary, T cell receptor signaling may be the primary pathway in the communication between gut and lung.

### Gut Microbiota–Bone Axis

The gut microbiota is responsible for bone physiology, and it can regulate bone mass via the immune system and promote bone resorption and formation via SCFA production (Ohlsson and Sjogren, 2015; Yan et al., 2016). In detail, the increased bone mass in germ-free animals was associated with a reduction in inflammatory cytokine expression in bone and less osteoclastogenesis. The change of the gut microbiota composition caused by dietary changes, antibiotic treatments or pathogens induces the imbalance in metabolic and immune regulatory networks, affecting bone mass (Ohlsson and Sjogren, 2015). Probiotics and prebiotics, especially *Lactobacillus* and *galactooligosaccharide*, regulate bone metabolism and promote bone growth by altering the gut microbiota composition and maintaining or increasing mass (Britton et al., 2014; Ohlsson and Sjogren, 2015; Schwarzer et al., 2016). *Lactobacillus* may reduce the expression of two inflammatory cytokines, TNF-α and IL-1β, increase the expression of osteoprotegerin (OPG), a potent inhibitor of osteoclastogenesis, and secrete beneficial immunomodulatory factors on ovariectomy-induced bone loss (Ohlsson et al., 2014). *Galactooligosaccharide* may improve calcium absorption and increase the relative proportion of bifidobacteria in the gut microbiota, possibly resulting in increasing bone mass (Whisner et al., 2013). The mechanisms involved the gut microbiota regulation of bone should be further investigated, since it might be a promising therapeutic theory for bone disease such as osteoporosis, osteoclastic bone resorption and rheumatoid arthritis.

## Gut Microbiota–miRNA Interactions in Physiology and Disease

Dysregulation of specific miRNAs has been found in many diseases including breast cancer (Iorio et al., 2005; Chin et al., 2016), gastric cancer (Li et al., 2015), non-small cell lung cancer (Yanaihara et al., 2006), and numerous inflammation-related disease and cancers (Lujambio and Lowe, 2012). Both microbiota and miRNAs play key roles in health and disease. The gut microbiota-miRNA interactions comprise two processes: (i) host-secreted miRNAs regulate the gut microbiota; (ii) the gut microbiota affects the host via inducing special miRNAs (Belcheva, 2017). These physiological processes are linked to host health.

### Gut Microbiota–miRNA Interactions and Intestinal Epithelia

**FIGURE 1:**
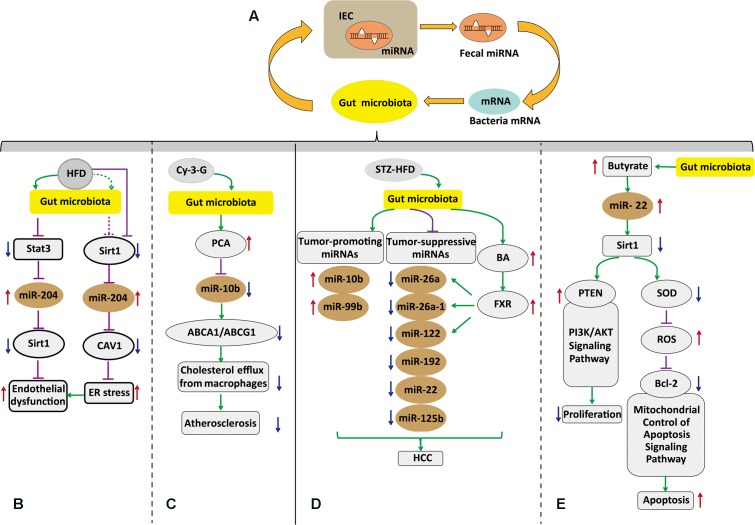
Host-secreated miRNAs regulate the gut microbiota that in turn affects the host via induction of specific miRNAs. **(A)** Gut microbiota–miRNAs interactions in intestinal epithelial. Fecal miRNAs shape the gut microbiota and then the gut microbiota has effects on miRNAs in IEC. **(B)** Postulated pathway through which the gut microbiota governs vascular endothelial dysfunction via altertion of miR-204. **(C)** The gut microbiota regulates macrophage cholesterol efflux and atherosclerotic lesion via down regulation miR-10b. **(D)** The gut microbiota contributes to HCC with sex-based disparity mediated by BAs and microRNAs. **(E)** SCFA butyrate from microbiota induced apoptosis and inhibited proliferation through up-regulation of miR-22 and repressing Sirt-1 expression in hepatic cells. IECs, intestinal epithelial cells; HCC, hepatocellular carcinoma; BAs, bile acids; Bcl-2, B-cell lymphoma-2; FXR, farnesoid X receptor; SCFA, short chain fatty acids; HFD, high-fat diet; STZ-HFD, streptozotocin-high fat diet; Cy-3-G, cyanidin-3-*O*-β-glucoside; Cav1, caveolin-1; PCA, protocatechuic acid; PTEN, phosphatase and tensin homolog; PI3K/AKT, phosphatidylinositol 3-kinase/protein kinase B; ABCA1, ATP-binding cassette transporter A1; ABCG1, ATP-binding cassette transporter G1; ROS, reactive oxygen species; SOD, superoxide dismutase. Pathway diagram key: 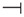 inhibition; →induction; 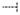 possible inhibition, ⇢possible induction; ↑upregulation; ↓downregulation.

The intestinal epithelial cells (IECs) are folded monolayer of cells that are exposed to the intestinal lumen (Gerbe et al., 2012). They play essential roles in nutrient absorption and hormone production, maintaining intestinal homeostasis (Peck et al., 2017). Moreover, when the microbiota communicates with other tissues, the intestinal epithelium is the first transmission channel for gut microbial signals (Schroeder and Backhed, 2016). Fecal miRNAs in gut lumen were partly from IEC. These miRNAs target bacterial mRNA, and then the host controls the gut microbiota via bacterial mRNA degradation or translational inhibition (**Figure 1**) (Liu et al., 2016). Liu et al. (2016) found that human miR-515-5p could target 16S rRNA/23S rRNA of *Fusobacterium nucleatum* and that miR-1226-5p could affect the yegH gene of *Escherichia coli* (Liu et al., 2016). Also, *Fusobacterium nucleatum* and *Escherichia coli* have been previously found to drive CRC (Rubinstein et al., 2013; Liu et al., 2016). However, the mechanisms by which miRNAs enter bacteria and interfere with specific mRNA transcription are not clear. A recent report showed that miRNAs could possibly enter the bacteria within endocytosis in *in vitro* experiments (Zhao et al., 2017). Delivery of miR155/let7g not only altered the microbiota, but also influenced cardiovascular function (Zhao et al., 2017). Therefore, it could be believed that these intestinal miRNAs from IEC or other tissues might shape the gut microbiota and then induce dysbiosis. Peck et al. (2017) have confirmed that the microbiota regulates miRNA expression in IEC subtypes, and the regulation may alter intestinal homeostasis. Quantified miRNA analysis among germ-free, conventionalized and conventionally raised chow-fed animals was achieved by miRquant. It was found that expression of some miRNAs are different among IEC subtypes and the difference depends on microbial status. Among these miRNAs, 19 miRNAs in intestinal epithelial stem cell (IESC) were significantly different. MiR-375-3p was the most activated, and only sensitive to the microbiota from IESC rather than other IEC subtypes. When miR-375-3p was knocked down, IESC proliferation increased (Peck et al., 2017). Similarly, Nakata et al. (2017) found that commensal bacteria induced the expression of miR-21-5p in IECs. However, there is no direct evidence showing how the microbiota regulates miRNAs. Possible mechanisms are based on the regulation of bacterial endotoxins and some metabolites or immune or mesenchymal cells signaling. As discussed above, fecal miRNAs from IEC affect bacterial gene expression, and the microbiota controls some miRNA expression in IESCs (**Figure 1**). These detailed mechanisms by which miRNAs participate in mediation of the gut microbiota and how microbiota affects miRNAs in IEC remain to be further investigated.

### Gut Microbiota–miRNA Interactions and Liver Disease

The gut microbiota promotes or suppresses liver disease, where, part of the alterations were attributed to the interaction between microbiota–miRNAs. Xie et al. (2017) reported that the gut microbiota is implicated in bile acid metabolism and miRNA expression to induce HCC with sex-based disparity (**Figure 1**). Differential expression of bile acid synthesis, transport genes, and differential accumulation of hepatic bile acid between female and male mice resulted in a sex-dependent incidence of liver carcinogenesis (Xie et al., 2017). Also, these altered miRNAs could possibly interfere with other signaling pathways affecting homeostasis. Further studies are needed to explore the signaling molecules from the gut microbiota to miRNAs and bile acid, and may provide a novel perspective to treat HCC with sex-based disparity.

Butyrate produced by the microbiota *in vivo* is involved in numerous functions of host physiology, including tumor suppression. The mechanisms by which butyrate suppresses tumor progression are different depending on the type of cancer cells (Stilling et al., 2016; Bultman, 2017). Pant et al. (2017) reported that the role of microbiota–miRNAs was linked by butyrate in hepatic cells. They found that butyrate upregulated miR-22 expression, followed by SIRT-1 downregulation, resulting in hepatic cell apoptosis. Although the proposed mechanism needs to be confirmed *in vivo*, it supplied valuable information for HCC treatment.

## Prospects

The gut microbiota is emerging as a crucial keeper of host’s health. Altered gut microbiota has been linked to various diseases. However the mechanisms by which the gut microbiota affects host’s health are poorly known. For example, further *in vivo* studies need to be performed to confirm the function of the gut microbiota-mediated miR-22 expression in liver cancer. Insight into the mechanisms of CNS diseases based on the microbiota gut–brain axis will provided a new research direction for studying CNS diseases. In addition, the gut microbiota, directly or indirectly, maintains homeostasis in many organ systems, but the mechanisms remain to be studied in detail. To conclude, the gut microbiota has physiological and pathological functions. Further investigation of the physiological function of the gut microbiota may supply promising and effective treatments in complex diseases.

## Author Contributions

QF wrote the manuscript. Y-DW and W-DC edited and revised the manuscript.

## Conflict of Interest Statement

The authors declare that the research was conducted in the absence of any commercial or financial relationships that could be construed as a potential conflict of interest.
